# Clinical, Echocardiography, biochemical and MRI findings in patients with acute left ventricular dysfunction to evaluate short-term outcome - A retrospective study

**DOI:** 10.6026/973206300213899

**Published:** 2025-10-31

**Authors:** Deen Dayal Nagar, Varsha Shrivastava, Yamini Batham

**Affiliations:** 1 Department of Pediatric Cardiology, Sawai Man Singh Medical College and J K Lone Hospital, Jaipur, Rajasthan, India; 2 Department of Pediatric Cardiology, Fortis Escorts Heart Institute, New Delhi, India; 3 Department of Pediatric Cardiology, Sri Satya Sai Sanjeevani Hospital, Palwal, Haryana, India

**Keywords:** Cardiomyopathy, LV dysfunction, LV Ejection Fraction, Congenital heart diseases, 2D-ECHO, Cardiac MRI

## Abstract

Heart failure in children is rare, but it strikes younger children at a younger age. Caregivers need training owing to the increased risk of death and the clinical presentation differs from that of adults. In order to forecast the early results in individuals with acute left ventricular dysfunction, it is necessary to assess several clinical, echocardiographic, biochemical and cardiac magnetic resonance imaging (MRI) results. The research took place in New Delhi at the Fortis Escorts Heart Institute's Department of Paediatric Cardiology. The research recruited 60 instances of severe acute LV dysfunction in children and adolescents (aged 1 month to 18 years) using a retrospective approach. Older age at admission, children with low LVEF on admission, requirement of ionotropic support on admission, Intracardiac thrombus on echocardiography evaluation and longer hospital stay were poor predictors of short-term outcome.

## Background:

Children with heart failure (HF) may have symptoms as early as birth or acquire the condition at any point during childhood. There are several causes of HF. In affluent nations, the most common are congenital heart disorders and primary cardiomyopathies (CMs), which together account for 60% of children who need a heart transplant [1, 2]. Primary CM has an incidence ten times greater in the 0-1 year old age group compared to the 0.8-1.3 cases per 100,000 children in the 0-18 year age group in industrialized nations [3, 4]. Due to the rarity of paediatric HF, the majority of doctors and nurses working in primary care and emergency rooms have never seen a case of it or dealt with a child with it. It is expected that the clinical manifestations may vary greatly and typically differ from adult symptoms. It is crucial to address the major challenges of early diagnosis and effective treatment for new-onset HF because 87% of these cases are only diagnosed when the patient is in a state of severe decompensation and less than 50% of children with symptoms of HF survive for 5 years without a heart transplant [5, 6]. Congenital heart disease (CHD) and cardiomyopathy are the leading causes of cardiac failure in children. According to Hsu and Pearson, HF in children is a clinical and pathophysiological syndrome that develops over time due to both cardiovascular and non-cardiovascular abnormalities. The symptoms of this syndrome include swelling, difficulty breathing, stunted growth and intolerance to physical activity. Additionally, there are molecular, hormonal and circulatory abnormalities that can be used to predict mortality rates early on [7].

Multiple methods exist for evaluating heart function. Systolic and diastolic function, as well as global and regional function of the left ventricle, may be evaluated using echocardiography. Volumes such as cardiac output, pressures, left ventricular mass and dP/dt may be measured. The wall motion score is another tool that may be used to evaluate dysfunction in certain regions [8]. The most reliable and non-invasive method for measuring the mass, function and volume of the left ventricle (LV) are cardiovascular magnetic resonance (CMR) imaging. With little inter- and intra-observer variability, it offers precise and repeatable volumetric evaluation of the left and right ventricles, as well as their diameters, volumes, wall thicknesses, mass and ejection %. Particularly when comparing echocardiography to radionuclide ventriculography and cardiovascular magnetic resonance, one can see that the three modalities' measurements differ significantly from one another [9, 10]. Assessment of heart function and stress may be aided by blood examinations, which play a crucial role in planning. Because they are not often detected in the bloodstream and because they give a high degree of clinical sensitivity and specificity even when cardiac lesions are modest, cardiac troponins in blood are the preferred indicators of myocardial injury [11]. Improved risk prediction seems to be achieved by measuring natriuretic peptides in conjunction with other well-established biomarkers of chronic HF, such as cardiac troponins (cTn) and hs-CRP, which suggest inflammation and myocardial necrosis, respectively [12, 13]. There is a large body of research supporting the use of cardiac Tn levels for risk stratification in individuals with acute and chronic HF. Generally speaking, the degree of decompensation and more severe illness are strongly correlated with rising cTn levels [14]. Clinical characteristics of LV dysfunction can vary from patient to patient. Some of them present with chief complaints of cough, fever, general weakness, syncope, chest pain, or tightness and signs and symptoms of heart failure. Severe cases have a history of shortness of breath and palpitations. Therefore, it is of interest to evaluate different clinical, echocardiographic, biochemical and cardiac MRI findings to predict the early outcomes in patients with acute LV dysfunction.

## Materials and Methods:

The researchers in this retrospective observational study gathered their data from June 2021 through July 2022 at the tertiary care facility in New Delhi known as the Fortis Escorts Heart Institute's Department of Pediatric Cardiology. The number of patients hospitalized in the Pediatric Cardiology Department at the Fortis Escorts Heart Institute in New Delhi for acute onset of left ventricular failure between January 2016 and December 2020 was used as the sample size. Inclusion in the trial was contingent upon the following criteria: age range of 1 month to 18 years, diagnosis of new-onset acute left ventricular dysfunction using 2D echo (LVEF < 30%) and clinical manifestation of sudden worsening of left ventricular function or pre-existing dysfunction as a result of cardiomyopathy or myocarditis. The research did not include patients who had valvular heart disease, congenital heart disease, or an inadequate transthoracic echocardiographic acoustic window, all of which may lead to left ventricular dysfunction. In order to gather data, previous patient records were examined. Information from inpatient medical records was gathered using a standardized data collection proforma. A thorough evaluation of the patient's hospital stay, including any treatments or complications, was conducted by reviewing their inpatient medical records. This included information regarding the patient's age, presenting symptoms, diagnosis, investigation, systemic examination (focusing on the heart), 2D echocardiography records and cardiac MRIs. Participants were included in the trial if they had severe heart failure (LVEF <30%). We enrolled 60 patients who met all inclusion and exclusion criteria. Data obtained from each patient included demographics (name, age, sex, weight, height), presenting symptoms, vitals & physical examination findings (Heart rate, Respiratory rate, looked for respiratory distress and features of poor perfusion and shock, pallor, cyanosis, oedema, clubbing, enlarged lymph node and temperature), different biochemical lab investigation results, treatment received and outcomes & complications. Poor peripheral perfusion was defined as a capillary refill time (CRT)>3 seconds. Rhythm was assessed from 12 12-lead ECGs and divided into sinus rhythm and non-sinus rhythm. All arrhythmias were classified as non-sinus rhythms. In order to categorize the stages of heart failure in children less than six years old, the Modified Ross classification was used, whereas the New York Heart Association (NYHA) classification was employed for children older than six years old. [As stated in the B-annex] On admission, a general physical examination was performed and the results, as well as any systemic examination results, were documented using the appropriate form. Left ventricular ejection fraction, fractional shortening, chamber size, mitral regurgitation and pericardial effusion were all measured using 2D echocardiography parameters. A left ventricular ejection fraction (LVEF) less than 30% was considered severe left ventricular systolic dysfunction. When the left ventricle diastolic and systolic diameters were more than 2 on the body surface area Z score, it was considered chamber dilation (LV). Severity grading of mitral regurgitation- mild, moderate, severe was defined using the heart valve disease module 4(BJC) - mitral regurgitation. Biochemical investigation results were collected retrospectively from records. Cardiac MRI findings were collected from the records and findings noted regarding involvement of ventricular myocardium for subtle or obvious changes of edema or scarring to diagnose non-ischemic myocarditis. Data on treatment given also includes anti-heart failure medications (Lasix, Envas, Carvedilol, Digoxin, Aldactone) and the need for ionotropic support during hospitalization and injectable Lasix. The ionotropic support requirement was divided into two groups. Ionodilator- dobutamine, milrinone and levosimendan while vasopressor- epinephrine, norepinephrine, dopamine and vasopressin.

## Statistical analysis:

The data were presented descriptively, with qualitative factors being represented by percentages and frequencies. "The median, 25th and 75th percentiles (interquartile range) and mean ± standard deviation were used to represent continuous variables that followed a normal distribution. The unpaired t-test was used to assess differences between groups. Numbers and percentages were used to represent categorical data and for comparing differences, either Fisher's exact test or the chi-squared test for trend was used. Microsoft Excel was used for data input and SPSS, or the Statistical Package for the Social Sciences, was used for the final analysis. A statistically significant result was defined as one with a p-value lower than 0.05."

## Results:

There was no gender bias with 30 (50%) males and 30 (50%) females. The majority of patients belonged to the age group >60 months (45%). Age was ≤ 12 months in only 19 out of 60 patients (31.7%). 6 patients were in the age group of 12-24 months and 8 patients were in the 24-60 months age group. The mean value of age (months) of study subjects was 71.1 ± 67.6 months, with a median of 48 months (Table 1 - see PDF). The most common presenting symptom was fast breathing (58.3%) and poor feeding (47.2%) followed by cough (25%), poor weight gain (16.7%) and fever (16.7%), diaphoresis(11.3%) and lethargy in younger children (0-6 years) while dyspnea (70.8%) and fatigue (62.5%) followed by palpitation (16.7%), abdominal pain (16.7%) and chest pain (12.5%) in older children (>6 years) (Table 2 - see PDF). The majority of older children [25% (n=15)], NYHA class was class II, followed by class III [11.7% (n=7)], while in younger children (<6 years), ROSS class was class III. [21.6% (n=13)] followed by class II [18.4% (n=11)] (Figure 1, 2 - see PDF). Out of 60 study subjects, 21.6% (n=13) patients had pallor and edema was present in 21.6% (n=13). 13.3% (n=8) were icteric. None of the patients had cyanosis or rash on admission. 21.7% (n=13) of patients out of 60 required some kind of assisted ventilation (CPAP/Mechanical ventilation) (Table 3 - see PDF). Mean value of LVEF by 2D echocardiography of study subjects was 23.3 ± 6.5 and CMRI LVEF was 20.1 ± 5.5. Median LVEF on 2D echocardiography and on CMRI was 25% and 20% respectively (Table 4 - see PDF). Mean value of MRI ESV (mL) of study subjects was 117.9 ± 87.01, with a median of 80.5. Mean value of MRI EDV (mL) of study subjects was 142.1± 96.5, with a median of 101.1 mL. Only three patients out of 60 study subjects had focal scaring on cardiac MRI evaluation, out of which 2 had mortality and one survived (Table 5 - see PDF). On 2D echocardiography evaluation, the majority of patients had mitral regurgitation. 56.7% (n=22) patients had mild MR, 13.3% (n=8) Moderate MR and 1.7% (n=1) had severe MR. Only 28.3% (n=17) of patients had no MR. All 60 (100%) patients had chamber dilatation (LA & LV). 8.3% (n=5) of patients had pericardial effusion. 8.3% (n=5) of patients found to have Intracardiac thrombus. In the majority [93.3% (n=56)] of patients, CPK-MB was within normal limits. CPK-MB was high in only 6.7% (n=4) of patients. In the majority (88.4%) of patients, Serum Calcium levels were within normal limits. Calcium was low in only 1.7% (n=1) of patients. 1 patient had an elevated serum calcium level on admission (1.7%). This patient was administered intravenous calcium in a previous centre, so the level was high. In the majority, 98.3% (n=59) of patients, Serum Magnesium levels were within normal limits. Magnesium was low in only 1.7% (n=1) of patients. In the majority, 73.3% (n=44) of patients, Serum Vitamin D levels were within normal limits. Vitamin D was low in 25% (n=15) of patients. 1.7% (n=1) of patients had an elevated Vitamin D level on admission. This patient was administered high doses of vitamin D in a previous centre, so the level was high. In the majority of 90% (n=54) of patients, the Thyroid function test was within normal limits. Hypothyroidism was present in only 10% (n=6) of patients (Figure 3 - see PDF). In 81.7% (n=49) of patients, Trop T levels were within normal limits. Trop T levels were elevated in 18.3% (n=11) patients.45 in 78.3% % (n=47) of patients, SGOT & SGPT levels was within normal limit. SGOT & SGPT levels were elevated in 21.7% (n=13) patients. Bilirubin levels were elevated in 13.3% (n=8) of patients. The majority of patients (78.3%, n=47) were non anaemic, while 21.6% (n=13) of patients had anaemia. Out of 21.6%, 8.3% (n=5) had mild, 10% (n=6) moderate and 3.3% (n=2) had severe anaemia. In the majority (90%, n=55) of patients, total leucocyte counts were within normal limits. TLC was elevated in only 8.3% (n=5) of patients. 1.7 %( n=1) patient had low TLC on admission. In the majority (95%, n=57) of patients, serum sodium was within normal limits. Serum sodium was low in only 5% (n=3) of patients. In the majority (95%, n=57) of patients, INR was within the normal limit. INR was deranged in only 5% (n=3) patients (Figure 4 - see PDF).

## Discussion:

In our study, we aimed to assess different clinical, echocardiographic, MRI and biochemical parameters in relation to short-term outcomes of patients with severe LV Dysfunction. We enrolled 60 study subjects retrospectively, collected their data and studied. The overall survival rate was 93.3% (n=56) while the mortality was 6.7% (n=4). Although the majority of other studies showed that children with severe LV dysfunction (dilated cardiomyopathy) present before their second birthday, whereas in our study, the mean age of admission was 71.1 months, with 41.7% children presenting before 24 months of age. The cause may be due to a delay in presentation or diagnosis in developing countries. Among the most notable clinical issues affecting children with severe LV dysfunction/cardiomyopathy is growth failure and low weight gain. Approximately one-third of these children may have growth failure to some extent while they are sick [15]. Forty percent of our participants were underweight for their age, while twenty percent were stunted. There was no correlation between malnutrition and unfavourable results in the statistical study. Fast breathing and poor eating were the most prevalent presenting symptoms in children aged 0-6 years, whereas dyspnea and exhaustion were the most common in children aged 6 years and above. Congestive cardiac failure (CCF) was evident at presentation with heart failure class III-IV in the majority of children; this was true for 65% of children aged 0-6 and 42% of children aged 6 and above. In contrast, the Indian researchers Anita *et al.* and Greenwood *et al.* found that every single patient in their study had CCF. A bad outcome is strongly related to a NYHA class IV and ROSS class IV upon presentation. There is strong evidence from previous research that suggests a strong correlation between the intensity of symptoms at presentation and a bad outcome [16, 17]. Of the participants in our research, 18.3% had an increased troponin T level; in the group that was readmitted or died, 22.2% (n=4 out of 18) had an elevated Trop T level, whereas 16.7% (n=7 out of 42) did not (p value 0.7). Up to 65% of the study group had increased troponin T levels, according to Rodriguez-Gonzalez *et al.* Thus, elevated troponin T levels in children may support the clinical suspicion of myocarditis and acute left ventricular failure, but they do not provide a conclusive diagnosis, as shown in our research. It is also recommended that, to prevent serious adverse effects, early detection is key for monitoring and initiating supportive therapy [18]. With CPK-MB, a similar finding was made. According to our findings, 3.3% of the 18 participants in the readmission/death group and 2.4% of the 42 participants in the no-death/no-readmission group had higher CPK-MB levels, with a Value of p = 0.07. Cardiac hepatopathy is indicated by abnormal transaminase values. These results suggest that circulatory failure may be linked to congestive hepatopathy and/or abrupt cardiogenic liver damage [19]. An increased transaminase level was observed in around 22% of the individuals in our research.

When diagnosing and evaluating individuals with severe left ventricular failure, echocardiography is a crucial tool. Our investigation revealed that the average left ventricular ejection fraction (LVEF) as measured by 2D echocardiography was 23.3% ± 6.5. Echocardiographic evaluation of left ventricular ejection fraction (LVEF) was significantly associated with outcomes in our research. The difference between the two groups in terms of LVEF was statistically significant (P = 0.01), with the former having a value of 20.5% and the latter of 25.0%. This confirms what previous studies have shown, for example, that a low LVEF is a strong independent predictor of death in a group of 47 pediatric patients from the Italian Trieste Heart Muscle Disease Registry. It is worth considering that high-risk individuals might benefit from increased diagnostic MRI use rather than endomyocardial biopsy [20, 21]. Some studies have shown that the degree of fibrosis predicts all-cause mortality and the need for future hospitalization [22]. Assomull *et al.* conducted a prospective longitudinal study to study the prognostic implications of midwall fibrosis in dilated cardiomyopathy (DCM). Their findings may have important prognostic implications [23]. In our study, all the study subjects underwent cardiac MRI to delineate the cause of LV dysfunction, ejection fraction and volumes of the chambers. Special attention was given to the cause to look for the changes of myocarditis (evidence of myocardial edema on cardiac MRI). We observed that decompensated shock, mechanical ventilation and multiple ionotropic support were the symptoms most strongly linked to a poor prognosis in children admitted to the cardiac intensive care unit with severe left ventricular dysfunction. By categorizing these cases as having a higher risk of mortality and morbidity, we can rationalize aggressive treatment. With a lower threshold for mechanical ventilation and more prudent use of inotropic agents, it might be further justified. Our research may lead to a better way of treating these people medically when ventricular assist devices and heart transplants aren't options.

## Conclusion:

The data indicate that children with severe left ventricular dysfunction (idiopathic dilated cardiomyopathy/myocarditis) have a poor prognosis. However, early transplantation could lower mortality rates for children who present more than 2 years after the diagnosis and who do not show improvement in echocardiographic indices, especially in cases where complex ventricular arrhythmias are diagnosed and mural thrombus is present. After echocardiographic parameters are back to normal, survivors are no longer safe against sudden death.
